# Editorial: Methods in avian physiology: 2023/24

**DOI:** 10.3389/fphys.2026.1649377

**Published:** 2026-05-21

**Authors:** Monika Proszkowiec-Weglarz, Joëlle Dupont, Elizabeth R. Gilbert

**Affiliations:** 1 Animal Biosciences and Biotechnology Laboratory, Agriculture Research Service, United States Department of Agriculture, Beltsville, MD, United States; 2 National Research Institute for Agriculture, Food and Environment (INRAE), Paris, France; 3 School of Neuroscience, Virginia Tech, Blacksburg, VA, United States

**Keywords:** avian, methods, birds, physiology, poultry

This Research Topic aimed to highlight the latest and up-to date experimental methods and techniques employed to answer questions related to all aspects of avian physiology, from molecular and cell physiology to organ function in wild and domestic birds. The goal was to provide a comprehensive overview of the current methods used to study avian physiology, highlighting their advancements, applications, and limitations. Contributions spanned a wide range of topics, including but not limited to avian genomics, transcriptomics, proteomics, metabolomics, the microbiome, physiology of avian cells and organ systems, metabolism, endocrinology, reproduction, nutrition, growth, and migration.

The Research Topic features studies on diverse subjects such as muscle satellite cell assays, non-invasive detection of broiler femur head lesions, anesthesia protocols in pigeons, tissue specific gene expression via recombinant adenovirus type-5 vectors, validation of apical-out enteroid *in vitro* models, intestinal permeability markers, and characterization of endogenous mucosal phosphatase.

Numerous studies have been published focused on the essential role of satellite cells in post-hatch skeletal muscle growth, and the myofiber repair and regeneration. Velleman in her opinion paper discussed different methods to study avian muscle satellite cells. She concluded that while all reviewed techniques are suitable to study biological properties of avian satellite cells isolated from pectoralis major, the choice of method should be guided by available resources and specific experimental objectives.

Ramser et al. discussed potential non-invasive approaches to detect femoral head lesions in broilers – a significant welfare and economic concern in poultry production. Traditional methods rely on visual assessment of leg and gait scores, and bone scoring during necropsy. In this article, the authors propose using dual-energy X-ray absorptiometry (DXA) imaging to identify necrotic regions in femurs.

Serir et al. developed a safe and effective anesthesia protocol for avian species, using pigeons as a model. By combining injectable and inhalational anesthetics, they established a protocol suitable for prolonged surgical procedures, which could be adapted for other bird species.

The paper by Choi et al. focused on determination of the optimal stage of chicken embryonic development for gene transfer into specific tissues via injection of recombinant adenovirus type 5 (Ad5) into blood vessels. They found that injecting Ad5 containing green fluorescent protein at Hamilton-Hamburger stages 14–17 of embryonic development resulted in heart-specific expression.

The next three papers focused on the gastrointestinal tract in chickens. Mann et al. validated a novel apical-out enteroid model for studying nutrient transport and gut barrier function, particularly under inflammatory and oxidative stress conditions. Efficiency of iohexol as an intestinal permeability marker was evaluated by Calik et al. in broiler chickens challenged with *Eimeria maxima*, *Clostridium perfringenes* or combination of both. The authors showed that indeed iohexol could be used as marker of intestinal permeability, which is important for the poultry industry given that impaired intestinal integrity in broiler chickens is associated with decreased performance and health. In the last manuscript by Hanauska et al. characterized in laying hens endogenous chicken mucosal phosphatase, an enzyme responsible for degradation of feed phytate. An enzyme activity assay and modified *in vitro* phytic acid (*myo*-inositol 1,2,3,4,5,6-hexakis (dihydrogen phosphate) degradation assay were employed in this study.

